# Metastasis-Specific CpG Island DNA Hypermethylation of the Long Non-Coding RNA Gene *00404* in Renal Cell Carcinoma

**DOI:** 10.3390/cancers17132204

**Published:** 2025-06-30

**Authors:** Pouriya Faraj Tabrizi, Inga Schimansky, Inga Peters, Jörg Hennenlotter, Hossein Tezval, Markus Antonius Kuczyk, Jürgen Serth

**Affiliations:** 1 Department of Urology and Urological Oncology, Hannover Medical School, 30625 Hannover, Germany; farajtabrizi.pouriya@mh-hannover.de (P.F.T.); tezval.hossein@mh-hannover.de (H.T.); kuczyk.markus@mh-hannover.de (M.A.K.); 2 Department of Hematology, Hemostaseology, Oncology and Stem Cell Transplantation, Hannover Medical School, 30625 Hannover, Germany; schimansky.inga@mh-hannover.de; 3 Department of Urology, Krankenhaus Nordwest, 60488 Frankfurt, Germany; peters.inga@khnw.de; 4 Department of Urology, Eberhard Karls University Tübingen, 72076 Tübingen, Germany; joerg.hennenlotter@med.uni-tuebingen.de

**Keywords:** renal cell carcinoma, DNA methylation, epigenetics, long non-protein-coding RNA

## Abstract

**Background/Objectives:** Alterations in long non-protein-coding RNAs (lncRNAs) are known to influence cellular proliferation, apoptosis, and metastasis in human cancers, including renal cell carcinoma (RCC). **Methods:** Using pyrosequencing, we analyzed DNA methylation (DNAm) at 23 loci within the *LINC00404* CpG island across 28 human cancer cell line models, 181 RCC tumor tissues, 154 paired tumor-adjacent normal tissues (adNs), and 194 metastatic tissue samples. **Results:** Our analysis revealed that all CpG sites exhibited tumor-specific hypermethylation (all *p* ≤ 1.4 × 10^−5^). Moreover, primary RCC tissues with distant metastases (M1) and metastatic tissue samples (Mtx) showed significant hypermethylation compared to RCC without distant metastases (M0). Notably, DNAm in Mtx displayed a significant increase in 22 CpG sites, compared to 12 CpG sites in the M1/M0 comparison, suggesting that DNAm in Mtx differs both qualitatively and quantitatively. **Conclusions:** Given that elevated levels of DNAm were also observed in the majority of cell line models, our findings suggest that *LINC00404* may play a pivotal role in the malignant development and progression of RCC metastasis, as well as in other human cancers.

## Simple Summary

Alterations in long non-coding RNAs (lncRNAs) are known to influence tumor biology in human cancers, including renal cell carcinoma (RCC). Here, DNA hypermethylation of CpG sites within the *LINC00404* gene in RCC is associated with advanced and metastatic disease, as well as with RCC metastases. These findings suggest that aberrant methylation of *LINC00404* may contribute to the development and progression of RCC.

## 1. Introduction

Long non-protein-coding RNA molecules (*lncRNAs*), microRNAs (*miRNA*), and pseudogenes are members of the non-coding RNA gene family that play a role in controlling important cellular functions like apoptosis, proliferation, epithelial–mesenchymal transitions, and metastasis in a variety of human cancers [1,2,3]. Changes in cellular proliferation, tumorgenicity, and disease progression in renal cell cancer (RCC) have also been associated with changes in the expression of both *lncRNAs* and pseudogenes [4,5,6]. So, biometric candidate analysis and confirmative experimental analysis for expression and sequence alterations suggested relevant roles of *lncRNAs* and pseudogene expression in RCC, showing a strong association between *lncRNA* alterations and the prognosis of RCC patients [7]. These RCC-specific findings are consistent with reports that a significant number of *lncRNAs* appear to be involved in direct regulation of metastasis in a wide range of human cancers [1].

RCC is one of the top ten most common cancers in the world, with an increasing incidence [8]. Given that a significant proportion of patients experience disease recurrence and metastasis after surgical treatment, and that the survival rate for patients with metastatic disease remains low, identifying molecular changes linked to the metastatic behavior of tumors is highly relevant because it may serve as a foundation for the identification of potential functional molecular targets and contributors to prognostic biomarker signatures [8,9,10,11,12].

The regulation of *lncRNA* expression has been described as highly complex. The activation of DNA methylases by *lncRNAs* for epigenetic silencing of target genes, for example, as well as the abnormal DNA methylation of *lncRNA* loci themselves, which results in hyper- and hypomethylation of *lncRNAs* affecting tumor suppressor or oncogene expression in cancer, respectively, have been reported [1].

The long intergenic non-protein-coding RNA 404 and 403 genes (*LINC00404*, *LINC00403*) are located close together on the long arm of chromosome 13, as are two CpG islands (CGIs) with 166 and 23 CpG sites, respectively. One of *LINC00403’s* two known gene transcript variants overlaps with the *SOX1* gene, also known as *SOX1* Overlapping Transcript (*SOX1*-OT) [13]. Changes in the expression of both transcripts have been linked to neural differentiation and have been observed in a variety of cancer cell line models (ibid.). However, corresponding analyses have not been reported for RCC cell lines.

Moreover, as far as we are aware, there has not been any published biomedical research focusing on *LINC00404* yet. Nonetheless, it has been included in a biometric signature that aims to identify mRNAs that are differentially expressed after a putative interaction between micro- and *lncRNAs* in lung and breast cancer [13,14,15]. Furthermore, changes in the DNA and the RNA expression of *LINC00403*, which is *LINC00404*’s immediate neighbor, have been documented and statistically correlate with patient clinical and pathological parameters, suggesting possible significance in the initiation and spread of cancer, e.g., whole exome sequencing identified a frameshift deletion in *LINC00403* in T-cell chronic lymphocytic leukemia [16], while lncRNA-microarray analysis for expression profiling showed that *LINC00403* RNA levels in esophageal cancer were reduced by approximately 8 times, among many other alterations [17]. Furthermore, it has been reported that *LINC00403* expression levels play a role in the biometrical classification of subgroups of uveal cancer that exhibit a greater likelihood of disease progression [18].

Notably, heavy smoking has been linked to changes in the DNA methylation of the CpG sites cg15653173 in *LINC00403*, cg24838345 in the *MTSS1*, and cg11068946 in the *NKX6-2* genes [19]. It is interesting to note that we recently discovered significant changes in the DNA methylation of *NKX6-2* loci, but in a different context of RCC metastasis [20]. Furthermore, similar to the methylation-based smoking signature, our biometric analysis of DNA methylation chip data revealed three candidate loci in the CGI located upstream of the *LINC00404* and *LINC00403* genes, the cg02742906, cg15415452, and cg13692446 sites, which show an association between methylation and the status of distant metastasis in RCC. As a result, the question arises whether the association of CGI methylation of the *LINC00404*/*00403* loci with metastatic renal tissues can be independently confirmed, providing rational starting points for both molecular analysis of RCC metastasis and association analyses of possible early lifestyle-induced alterations affecting tumor progression odds. Here, we compare the methylation of the CGI linked to the *LINC00404* and *LINC00403* genes in primary RCC samples (M0 and M1) and metastatic tissue samples (Mtx). The results show a strong statistical correlation between DNA methylation and kidney tissue metastatic status. Furthermore, loci displaying both qualitative and quantitative alterations in DNAm were found in samples of metastatic tissue.

## 2. Material and Methods

### 2.1. Study Design

To identify metastasis-associated candidate loci, DNA methylation (DNAm) profiling was carried out using an in silico analysis of level 3 data from the TCGA KIRC HM450k methylation dataset and the statistical software R v4.0, including the Bioconductor and minfi software packages as described previously [21,22,23]. Candidates were ranked by multiplying the negative decadic logarithm of *p*-values by the fold change in DNAm. Pyrosequencing-based methylation analysis was conducted using negative and positive controls, as well as human primary and tumor cell line models, as previously described [20]. The relative methylation values of normal, tumoral, and metastatic tissue samples were compared and statistically evaluated in a cross-sectional study.

### 2.2. Study Cohort

Methylation analyses were carried out in 181 RCC tumor tissues, 154 paired tumor adjacent normal tissues (adNs), and 194 metastatic tissues from 95 patients with metastatic RCC disease. The patient characteristics, metastatic tissue cohort, tissue sampling, TNM classification, grading, and tissue treatment were previously described [22,24,25]. Ethical approval was granted by the ethical boards of Eberhard Karls University Tübingen and Hanover Medical School (no. 128/2003V and 1213-2011, approved 14 October 2011). Written informed consent was obtained from all patients. The study was performed in accordance with the Helsinki Declaration.

### 2.3. Preparation of DNA and DNA Methylation Analysis

The histological estimation of tumor cell content, DNA isolation from frozen sections and formalin-fixed paraffin-embedded tissue sample punches, and bisulfite conversion of DNA were performed as previously described [26,27]. Pyrosequencing was used to analyze DNA methylation. PCR reactions and pyrosequencing template preparation have been described previously [26,28]. The *LINC00404* CGI pyrosequencing assays were designed by use of the PyroMark Assay Design 2.0 software (Qiagen, Hilden, Germany) and the hg19 genome assembly as provided by the UCSC table browser. Supplementary Table S1 contains the forward, reverse, and sequencing primer sequences, as well as the sequence to analyze for pyrosequencing.

### 2.4. Statistical Analysis

Statistical analyses were conducted using R v4.3.0 software, R-Studio^®^, and program libraries as specified below [23,29]. Statistical tissue group comparisons were carried out using CpG site-specific methylation values. Tumor-specific hypermethylation in paired samples was assessed using the two-sided paired *t*-test, while independent group comparisons were made using bivariate logistic regression models with age and sex as covariates. Multiple metastatic tissues were evaluated following patient-wise aggregation and the calculation of mean methylation values.

## 3. Results

### 3.1. Identification of Candidate Loci and Analysis of LINC00404/LINC00403 Associated CpG Island Methylation in Cell Line Models

**Figure 1:**
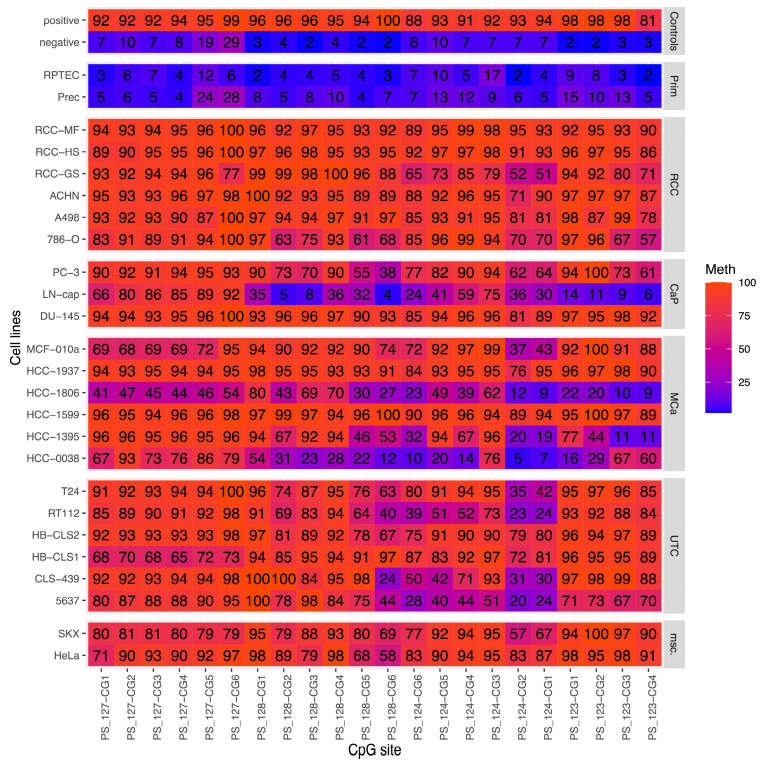
Heatmap showing DNA methylation analysis of artificial DNA preparations (controls), primary cells (Prim), renal cell cancer cell lines (RCC), prostate cancer cell lines (CaP), mammary cancer cell lines (MCa), urothelial cancer cell lines (UTC), and miscellaneous cancer models (msc). The relative methylation levels are represented by numbers and colors, from low (blue) to high (red). Note that the presentation of particular CpG sites follows their genomic order. Supplementary Table S2 shows the exact genomic positions of CpG sites. CpG site PS_127-CG7 is not displayed due to technical reasons (incomplete data).

In silico analysis of TCGA-KIRC data identified cg02742906, cg15415452, and cg13692446 as among the top 150 candidate loci (ranks 11, 138, and 89) for differential DNAm in RCC with M0 or M1 status for distant metastasis (Table 1). All of the candidates were part of the CGI on chromosome 13 (112,758,599–112,760,491), which has 166 CpG sites and is located upstream of the two *lincRNAs* 00404 and 00403. Supplementary Figure S1 depicts the genomic context of the *LINC00404* and *LINC00403* genes, the approximate location of candidate CpG sites, and DNA methylation of specific CpG sites in cancer cell line models. Supplementary Table S2 contains descriptions of the genomic positions of biometrical candidate sites and loci that can be accessed via pyrosequencing analysis and used for statistical evaluation. Our pyrosequencing analyses included four regions of the *LINC00404*/*LINC00403* associated CGI, totaling 23 CpG sites (Supplementary Table S2), and were first performed for 26 cell line models representing primary cells of the kidney and prostate (two cell models), RCC (6 cell lines), prostate cancer (3), breast cancer (8), urothelial cancer (7), and other malignancies (2). We found low to absent methylation in primary cells and overall medium to high methylation in cancer cells (Figure 1). Notably, RCC cell line models had a mostly highly homogeneous methylation of more than 90%, whereas prostate, breast, and urothelial cancer models showed a higher degree of heterogeneity (5–100% methylation).

### 3.2. Hypermethylation of the LINC00404 and LINC00403 CGI in Renal Cell Cancer

**Figure 2:**
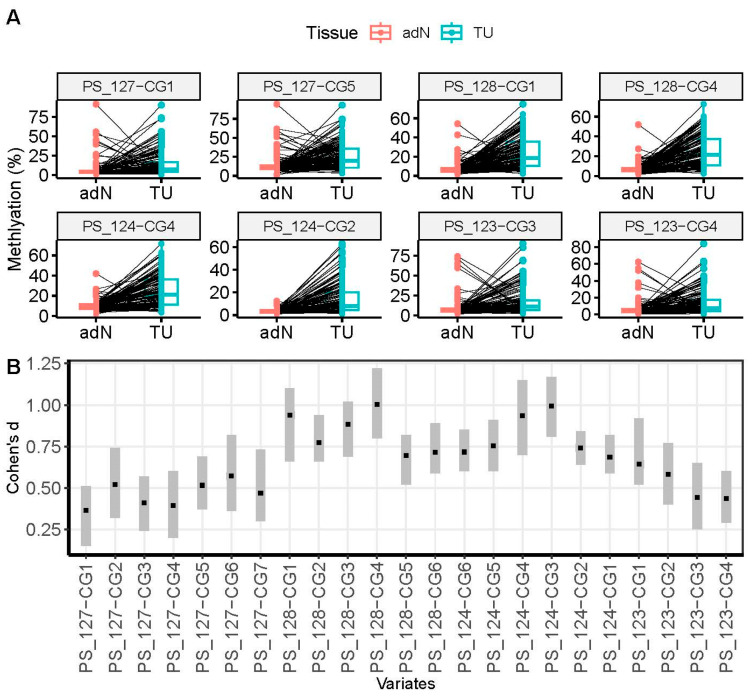
(**A**) Presentation of paired tumor-specific hypermethylation (black lines) of exemplary CpG sites as acquired for tumor tissues (TU, turquoise dots) and normal tumor adjacent tissues (adN, apricot dots). Supplementary Table S3 gives statistical results for all CpG sites measured. (**B**) Effect size presentation using Cohen’s d analysis for all CpG sites measured in genomic order. The value of Cohen’s d is depicted in black squares with confidence intervals indicated by grayed bars. Supplementary Table S4 displays Cohen’s d values, confidence intervals, and effect sizes for each measured CpG site.

We used pyrosequencing to analyze the methylation of 23 CpG loci in four regions of the *LINC00404* and *LINC00403* CGI in 154 tissue pairs of adN and tumoral tissue samples. All CpG sites exhibited statistically significant tumor-specific hypermethylation (two-sided *t*-test, all *p* ≤ 1.4 × 10^−5^, Benjamini–Hochberg correction for multiple testing, Figure 2A and Supplementary Tables S2 and S3). Cohen’s d statistical analysis found large effects at five CpG sites (PS_128-CG1,~3, ~4; PS_124-CG4, ~3), moderate effects at 12 sites, and small effects at 6 sites (Figure 2B and Supplementary Table S4). Median ratios for tumor vs. paired adN tissue methylation ranged from 3.0 to 3.6 for the CpG sites with large effects.

### 3.3. Hypermethylation of the LINC00404/LINC00403 CGI in Aggressive Primary Cancers

**Figure 3:**
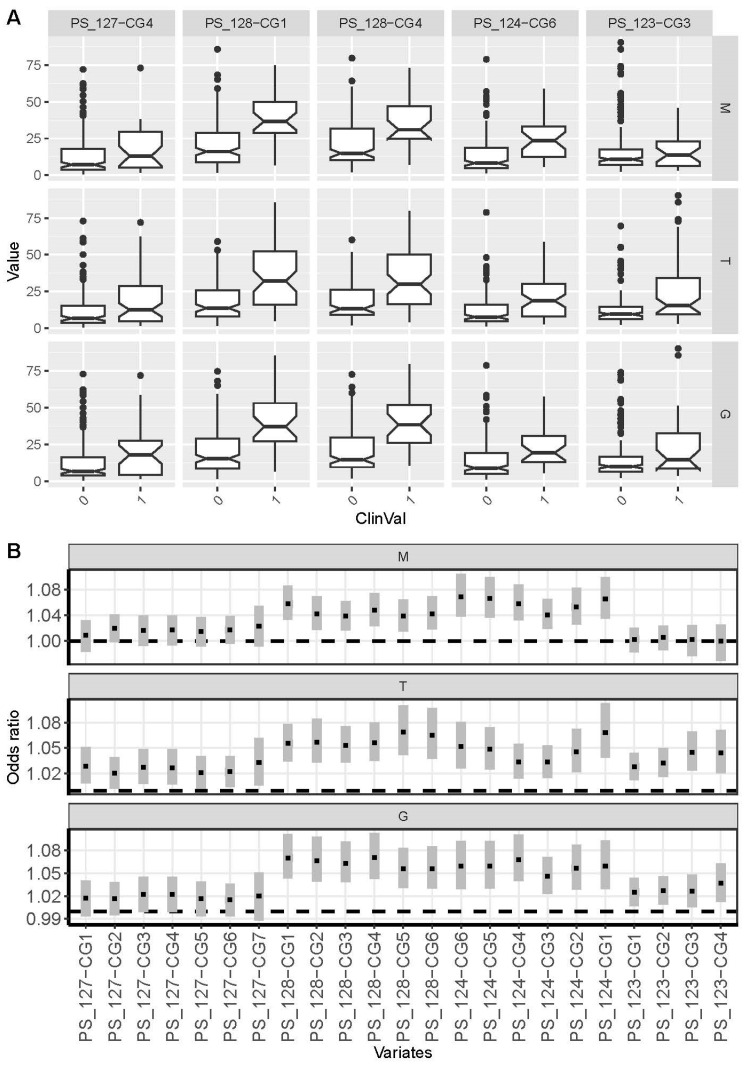
(**A**) Boxplot analysis of DNA methylation level distributions in primary RCC tissues stratified for the presence (1) or absence (0) of distant metastasis (M), high (≥T3) and low (<T3) tumor stages, and high (≥G3) and low (<G3) tumor cell differentiation for a subset of exemplary CpG sites. Medians, notches representing the estimated confidence interval, 25% and 75% quartiles, whiskers indicating the 99.3% interval (two-sided 1.5-fold of interquartile range), and outliers (black dots) of the relative methylation distributions are shown. (**B**) Forest plot presentation of CpG site-specific odds ratios and confidence intervals obtained by logistic regression analysis for tumor group comparisons as described in (**A**). Note that the presentation of particular CpG sites follows their genomic order. Supplementary Table S5 presents detailed results of logistic regression analysis of the parameter distant metastasis for all CpG sites analyzed.

The statistical analysis of the most important and available clinical parameters, including the presence of distant metastasis, low and high stage tumors, and low- and high-grade histopathological differentiation of tumor cells in total, revealed a significantly higher mean methylation for clinically more aggressive primary tumors. So, when tumors were stratified for the absence (M0) or presence of distant (M1) metastasis, M1 tumors had significantly higher mean methylation in 12 out of 23 CpG sites, as shown in the boxplot and logistic regression analysis in Figure 3A,B (each first row). The odds ratios obtained for the comparison of M0 and M1 tumors were between 1.04 and 1.07, corresponding to a 1% methylation change in tumors, and were observed predominantly in the central part of the CGI (PS_128-CG1–PS_124-CG1; Benjamini–Hochberg adjusted *p*-values between 1.18 × 10^−3^–4.6 × 10^−6^, Supplementary Table S5 and Figure 3B). Similar results were obtained for the analyses of high-stage and high-grade tumors, both in terms of the size of the effect expressed as an odds ratio and the genomic distribution of CpG sites in the central part of the CGI (Figure 3A and Figure 3B, second and third rows, respectively). Supplementary Figure S2A–C show boxplot analyses of all analyzed CpG sites. A comparison of tumor samples histopathologically diagnosed as papillary renal cancer (pRCC) and clear cell RCC (ccRCC) or tumors with mixed histological appearance indicated a potential relevance of the PS_128-CG5 and ~6 CpG sites showing potentially decreased methylation in pRCC without reaching statistical significance after correction for multiple testing (Supplementary Figure S2D). The covariates age and sex, though demonstrating possible contributions for the association of some CpG sites with the status of distant metastasis and grading of tumors, likewise did not show up with statistical significance following adjustment for multiple testing.

### 3.4. Qualitative and Quantitative Alterations of the LINC00404 and LINC00403 CGI Methylation in Metastatic Tissue

**Figure 4:**
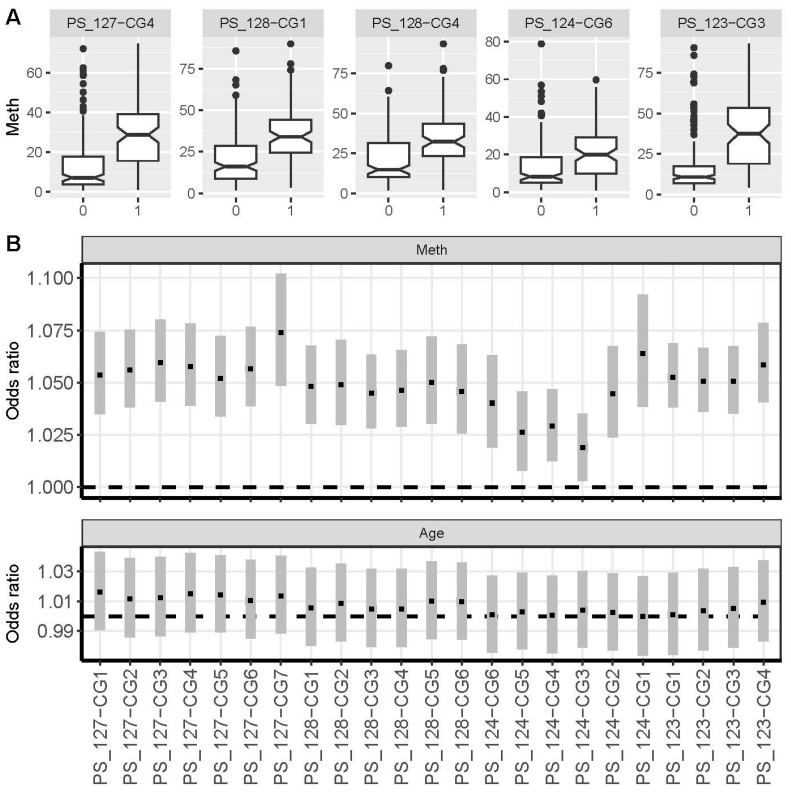
(**A**) Boxplot analysis of DNA methylation level distributions for primary RCC without distant metastasis (0) and metastatic tissues (1) for a subset of exemplary CpG sites. Boxplot presentation as described in Figure 3A. (**B**) Forest plots depicting the results of multivariate logistic regression analysis of all measured CpG sites, including the target variable methylation (Meth) and the covariate age. Supplementary Table S6 shows the detailed results of the logistic regression analysis of primary RCC without distant metastasis and metastatic tissues at all CpG sites examined.

The comparison of DNA methylation of loci in RCC samples in the M0 state and tissue samples obtained from RCC metastatic tissues revealed that all but one of the loci (PS_124-CG3) are significantly hypermethylated in metastases (Figure 4A,B and Table 2). When comparing the medians of DNAm in tumors of M0 state and metastatic tissues, distant metastases showed up to a 4-fold increase in methylation (PS_127-CG1, PS_127-CG4, Supplementary Table S5). This finding is roughly consistent with the results of our logistic regression analysis, which revealed odds ratios of approximately 1.04–1.075 per 1% methylation increase in metastatic tissue samples for the parameter DNAm (Figure 4B). Given that the median methylation of the PS_128_CG1 locus differs by 14% between M0 and metastatic tissues, the locus-specific odds ratio of 1.064 corresponds to odds of roughly 2.4. Additionally, our analysis demonstrated that in logistic regression models, sex and age were not significant parameters.

**Figure 5:**
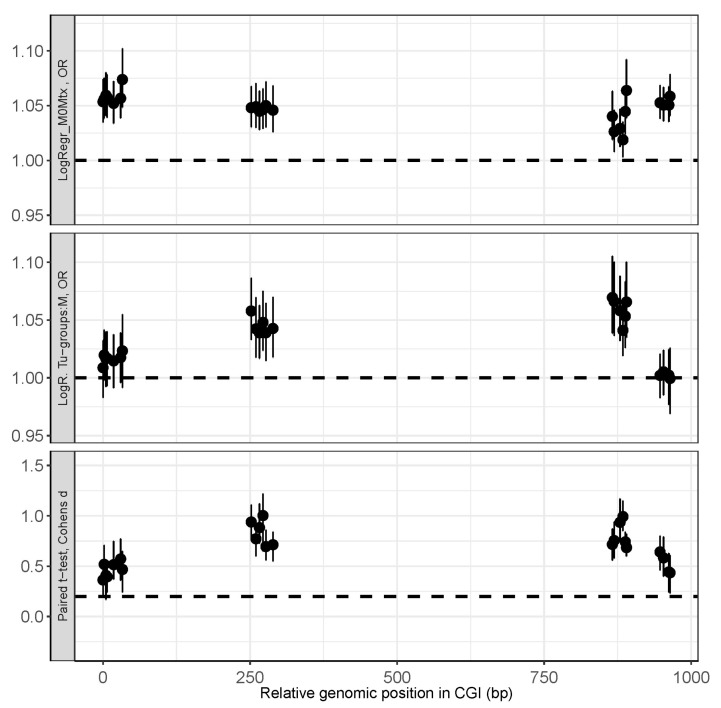
Comparison of statistical effects of methylation status in metastatic tissues, primary tumor tissues, and adjacent normal tissues (adN), depending on genomic location. Top panel: Comparison between M0 tumors and metastatic (Mtx) tissues reveals statistically significant hypermethylation at loci at the 5′-end of the CGI, as well as at loci approximately 900 bp downstream within the CGI. Middle panel: Comparison between tumor groups shows weak or marginally statistically significant effects. Bottom panel: Comparison of tumors and adN tissues shows weak or non-significant statistical effects. Note that statistical effect sizes depend on genomic location. The dashed line in the top and middle panels indicates an odds ratio (OR) of 1 (no effect). The dashed line in the bottom panel indicates a Cohen’s d of 0.2 (negligible effect).

Notably, the overall comparison of statistical effects obtained for tumor-specific hypermethylation (Cohen’s d), tumor-group comparisons (OR), and M0 tumor vs. Mtx tissue comparison (OR) revealed that loci at the 5′-end of the CGI as well as loci located about 900 bp downstream in the CGI exhibit specific methylation alterations in metastatic tissues (Figure 5). Both groups of loci show poor (tumor-specific hypermethylation, ibid, bottom panel) or no statistically significant effects (tumor group comparison, ibid, middle panel), while the comparison between M0 tumors and Mtx tissues reveals maximum effects (ibid, upper panels).

## 4. Discussion

Determining the molecular alterations linked to RCC progression can offer important information about the mechanisms behind the metastasis process. Moreover, molecular changes may serve as specific biomarkers indicative of metastatic progression, helping clinicians develop personalized treatment targeting aggressive tumor subtypes more effectively. Non-coding RNA gene family members, including *lncRNAs*, *miRNAs*, and pseudogenes, may have important roles in the prognosis of RCC patients, according to systematic biometric candidate analysis and confirmatory experimental analysis for expression and sequence alterations [30]. Furthermore, it is evident from the literature that a range of mechanisms, including epigenetic modifications like DNA methylation, control the expression of non-coding RNAs [30]. Previous analyses by others and our group identified DNA methylation alteration of several miR-genes occurring in RCC. So, we found association of DNAm of the *miR124-3* gene CGI with the metastatic state of RCC [27]. On the other hand, relatively little is known about the modification of *lncRNAs* by DNAm. In a previous biometrical analysis of TCGA methylation chip data, we identified CpG loci in the *LINC00404*/*00403* CGI as potential markers for the metastatic state of RCC. We then sought to provide independent evidence for the relevance of such alterations in human cancer tumor models as well as RCC and associated metastatic tissues. When DNA methylation was quantified using pyrosequencing analysis, it was shown that all measurable CpG sites—which span a significant portion of the CGI—had high relative methylation in all of the human cancer cell line models examined. Notably, RCC-derived cell lines showed the most uniform high relative methylation values, reaching up to 100% (Figure 1). Prostate, breast, and urothelial cancer groups also showed relatively high, albeit somewhat more variable, relative methylation values. Apart from the primary and negative control cells, the methylation of the LINC00404/00403 CGI appears relatively high overall, suggesting that methylation of this CGI is a shared feature of a variety of human tumor cells. Significantly, homogenous high methylation was also shown by the HeLa and SKX cell lines. This finding is similar to data for candidate sites reported by the ENCODE project and displayed in the UCSC table browser (Supplementary Figure S1) [27,31,32].The analysis of tumor-specific hypermethylation in paired renal tissues revealed a significant increase in tumor-specific methylation at all CpG sites studied, with large or moderate statistical effect sizes at five and twelve CpG sites, respectively. Thus, a strong hypermethylation effect in this CGI could be seen, with a maximum of a median 3.6-fold increase in methylation in tumors observed. These findings raise the possibility of functional implications, such as the epigenetic silencing of LINC mRNA expression. However, available data in the TCGA database indicate mRNA expression levels at the borderline of the detection limit, questioning the results of a biometrical in silico analysis of us, which indicated tumor-specific loss of *mRNA* expression (*p* < 0.05). Given that epigenetic mutual interaction of *lncRNAs*, *miRs*, and pseudogenes is also influenced by the effects of epigenetic modifications of histones and DNA, it appears that in-depth targeted analysis will be required to gain further insight into functional properties. According to the RNAinter database, functional analyses of the *LINC00404* gene may benefit from considering its interactions with histone modifications such as H3K27me1, H3K27me2, H3K27me3, and H3K27ac, as well as transcriptional regulation by factors such as CTCF, POU5F1, SOX2, TAL1, HNF4A, GATA4, and EZH2 [33]. Notably, the GenHancer database reports shared regulatory elements between *LINC00404* and other genes, including *SOX1*, *lnc-SOX1*-5, *SPACA7*, *HSALNG0099469*, *HSALNG0099470,* and *LOC124900341* [34]. Furthermore, modulation of *LINC00404* expression may occur via interaction with the microRNAs hsa-miR-141 and hsa-miR-200a [14]. In addition, the presence of an oxygen-responsive element (ORE) within the *LINC00404* sequence raises the possibility of regulation or modulation by known ORE-binding proteins such as RBPJκ, CXXC5, and MNRR1 [35].

Whether methylation marks in the *LINC00404*/*00403* CGI are associated with aggressive RCC was analyzed by comparison of primary cancers with varying state of distant metastasis (M0, M1) as well as by assessment of metastatic tissues obtained from RCC patients. Approximately half of the CpG sites examined showed significantly higher methylation in primary cancers with distant metastasis (Figure 3). Furthermore, plotting the ORs obtained from logistic regression analyses in the genomic order of the CpG sites analyzed revealed that significant alterations were all directly adjacent and followed the same pattern as the corresponding analysis of high-stage or high-grade tumors (Figure 3B). This applies to the CpG sites analyzed within a relatively narrow genomic region of chromosome 13, ranging from positions 112,759,087 to 112,759,725 (PS_128-CG1–PS_124-CG1), while neither behind position 112,759,782 (PS_123-CG1) nor before position 112,758,868 (PS_127-CG7) significant alterations could be observed (Supplementary Tables S2 and S5). Most intriguingly, the positional analysis of OR’s computed for the comparison of M0 tumors and metastatic tissue samples showed a remarkably different picture. Presumably, the hypermethylated region expands during RCC-derived metastatic tissue growth, as the entire region exhibits significantly increased methylation, including CpG sites that did not previously reveal M1 tissue-specific hypermethylation but now show the statistically most prominent effects (PS_127-CG1-PS_127-CG7, chr. 13, positions 112,758,835–112,758,868). This finding appears to be of high interest for the development of biomarkers for the detection of metastatic tissues, as it demonstrates the presence of both quantitative and qualitative differences in *LINC00404*/*00403* CGI methylation in RCC metastases. 

Therefore, the biometric identification of candidate loci indicative of metastatic RCC obtained from TCGA data is independently confirmed and expanded upon by our data regarding the patient cohort, tissue types investigated, and DNA methylation detection method.

While our statistical results cannot provide functional evidence for the relevance of *LINC00404*/*00403* in the process of RCC metastasis, they clearly offer a rationale for a promising starting point for deeper functional analyses. Hence, a wide range of *lncRNAs*, many of which are involved in invasion and EMT mechanisms, have been functionally linked to cancer metastasis [1,30]. Furthermore, it has been reported that the PTENP1 pseudogene in RCC exhibits epigenetic silencing of pseudogene expression, and that the tumor suppressor *lncRNAs MEG3* and *LOC554202* show loss of expression as a result of aberrant promoter methylation and are associated with cancer cell invasiveness (ibid.). Thus, assuming that loss of mRNA expression of *LINC00404*/*00403* is also linked to DNA methylation in corresponding studies, the findings of the current study support the relevance of such alterations for a significant proportion of cases with metastatic RCC.

In view of efforts to improve personalized treatment, also in metastatic RCC, improved biomarkers for early identification of aggressive cancer subtypes are required. We have recently described the first renal metastasis-associated methylation signature (RMAMS) [20] but have not yet included information about methylated loci in lncRNA genes.

Given that alterations to the *LINC00404*/*00403* CGI appear to occur frequently and provide metastatic tissue-specific information, we hypothesize that DNAm alterations in *lncRNA* genes could improve such biomarker profiles. Interestingly, despite the availability of extensive molecular databases, the relevance of *LINC00404*/*00403* and *lncRNA* alterations in human cancers remains unknown, and speculation about possible functional involvement in tumor-relevant signaling pathways appears to be impossible at this time. As a result, our findings emphasize the importance of further characterizing the role of these *lncRNAs* in RCC cancer metastasis as well as other human tumors in general.

## Figures and Tables

**Table 1:** Genomic positions and results of logistic regression analysis of biometric identification of candidate CpG sites of primary RCC showing distant metastasis.

Locus	chr	pos	Rank	*p*Val	OR	mn0	mn1	fold
cg02742906	chr13	112,758,625	11	5.12 × 10^−9^	28.15	0.20	0.45	2.30
cg15415452	chr13	112,759,355	138	3.48 × 10^−7^	14.35	0.21	0.44	2.13
cg13692446	chr13	112,759,719	89	6.93 × 10^−8^	27.37	0.21	0.44	2.09

Abbreviations: Chromosome (chr), position in bp (pos), *p*-value (*p*Val), odds ratio (OR), mean ß-values of RCC tissues without distant metastasis (mn0), mean ß-values for RCC with distant metastasis (mn1), fold change in ß-values between tissue groups (fold).

**Table 2:** Logistic regression analysis for tumor group comparison of RCC M0 and M1 tissues.

Var	OR	conf.low	conf.high	*p*.value	sig	*p*.adj	sig.adj
PS_127-CG1	1.054	1.035	1.074	0.000000	***	0.000000	***
PS_127-CG2	1.056	1.038	1.075	0.000000	***	0.000000	***
PS_127-CG3	1.059	1.041	1.080	0.000000	***	0.000000	***
PS_127-CG4	1.058	1.039	1.078	0.000000	***	0.000000	***
PS_127-CG5	1.052	1.034	1.072	0.000000	***	0.000000	***
PS_127-CG6	1.057	1.039	1.077	0.000000	***	0.000000	***
PS_127-CG7	1.074	1.048	1.102	0.000000	***	0.000000	***
PS_128-CG1	1.048	1.030	1.068	0.000000	***	0.000001	***
PS_128-CG2	1.049	1.030	1.070	0.000001	***	0.000007	***
PS_128-CG3	1.045	1.028	1.063	0.000000	***	0.000002	***
PS_128-CG4	1.046	1.029	1.066	0.000000	***	0.000002	***
PS_128-CG5	1.050	1.030	1.072	0.000001	***	0.000007	***
PS_128-CG6	1.046	1.026	1.068	0.000013	***	0.000064	***
PS_124-CG6	1.040	1.019	1.063	0.000256	***	0.001099	**
PS_124-CG5	1.026	1.008	1.046	0.005993	**	0.023398	*
PS_124-CG4	1.029	1.013	1.047	0.000681	***	0.002787	**
PS_124-CG3	1.019	1.003	1.035	0.020042	*	0.074842	.
PS_124-CG2	1.045	1.024	1.067	0.000038	***	0.000172	***
PS_124-CG1	1.064	1.039	1.092	0.000001	***	0.000007	***
PS_123-CG1	1.053	1.038	1.069	0.000000	***	0.000000	***
PS_123-CG2	1.051	1.036	1.067	0.000000	***	0.000000	***
PS_123-CG3	1.051	1.035	1.067	0.000000	***	0.000000	***
PS_123-CG4	1.059	1.041	1.079	0.000000	***	0.000000	***

Abbreviations: Variable (Var), odds ratio (OR), lower confidence interval (conf.low), upper confidence interval (conf.high), *p*-value (*p*.value), significance (sig), Benjamini–Hochberg adjusted *p*-value (*p*.adj), adjusted significance (sig.adj). Graphical presentation of significant levels: *: *p* < 0.05; **: *p* < 0.01; ***: *p* < 0.001; .: not significant.

## Data Availability

The original contributions presented in this study are included in the article/Supplementary Materials. Further inquiries can be directed to the corresponding author.

## Supplementary Materials

The following supporting information can be downloaded at: https://www.mdpi.com/article/10.3390/cancers17132204/s1, Table S1: Oligonucleotides used for pyrosequencing analysis. Table S2: Genomic location of candidate and CpG sites measured by pyrosequencing. Table S3: Statistical analysis of methylation in paired normal-adjacent and tumor tissues. Table S4: Cohen´s d analysis of paired normal-adjacent and tumor tissues. Table S5: Logistic regression analysis of primary RCC with and without distant metastasis. Table S6: Descriptive statistics of primary and RCC metastatic tissue groups. Figure S1. Genomic context of the *LINC00404* and *LINC00403* genes, location of the CpG islands, the approximate location of candidate CpG sites, and DNA methylation of specific CpG sites in cancer cell line models as reported by the ENCODE project. Figure S2. Box plot analysis of tumor group comparisons across all CpG sites following dichotomization: (A) presence or absence of distant metastasis (M), (B) tumor stage classified as high versus low (T), (C) tumor grade classified as high versus low differentiation (G), and (D) histological subtype, distinguishing papillary or clear cell, including mixed tumor histologies.
