# Supplementary material for: Metastasis-Specific CpG Island DNA Hypermethylation of the Long Non-Coding RNA Gene 00404 in Renal Cell Carcinoma
Source: Cancers (Basel). 2025 Jun 30;17(13):2204. doi: 10.3390/cancers17132204 (PMC12249281; doi:10.3390/cancers17132204)
Supplement: Supplementary file 1 [file cancers-17-02204-s001.zip › Tables.pdf]

Supplemental Table S1. Oligonucleotides used for pyrosequencing analysis.

Assay	Seq.Type	Seq.
PS_127	FW	AGTGGGGATAAGGGAGAAAGAGTA
PS_127	RV	5BioACCCTAAAACTAAATATCCATAACCT
PS_127	SQ	AAAGAGTAAGTTAGTAGG
PS_127	StoA	TTAYGYGGYGYGTGGGTAGTGYGTAATTTTTGTYGGYGTTTAGGTTGTA
PS_128	FW	GGGGTATAGGAAGGTAGTTTAAGAG
PS_128	RV	5BioCCCCCAACACCAAATTACTAATCC
PS_128	SQ	GTATAGGAAGGTAGTTTAAGAGT
PS_128	StoA	GTTYGTATGGTYGAGGAYGTTTTYGTTGYGAGTTTGGGGTYGAGGGGTAGTTTTTTT<br>T
PS_123	FW	GGGTAGGTGTGGGTTAGTT
PS_123	RV	5BioACACCAACACCTAAACAC
PS_123	SQ	GGTGTGGGTTAGTTT
PS_123	StoA	YGTTATYGTTTGAGAYGYGGTGTGTTTAGGTGTTGGTGT
PS_124	FW	5BioTTGGAAATTTGAGGTTGGATTAAT
PS_124	RV	AAAAACCTCTCCAAATCCCTACC
PS_124	SQ	TCCAAATCCCTACCC
PS_124	StoA	RCRTTCRCTACRAATTACAACRACRCTCATCTCRAAAATATAAC

Abbreviations: Type of oligonucleotide (Seq.Type), Sequence (Seq.), Forward primer (FW), Reverse primer (RV), Sequencing primer (SQ), Sequence to analyze (StoA), 5´-Biotinylation (5Bio)

Supplemental Table S2. Genomic location of candidate and CpG sites measured by pyrosequencing.

Assay_cg	Chr	Pos	Candid
	chr13	112,758,625	cg02742906
PS_127-CG1	chr13	112,758,835	
PS_127-CG2	chr13	112,758,837	
PS_127-CG3	chr13	112,758,840	
PS_127-CG4	chr13	112,758,842	
PS_127-CG5	chr13	112,758,853	
PS_127-CG6	chr13	112,758,865	
PS_127-CG7	chr13	112,758,868	
PS_128-CG1	chr13	112,759,087	
PS_128-CG2	chr13	112,759,095	
PS_128-CG3	chr13	112,759,101	
PS_128-CG4	chr13	112,759,107	
PS_128-CG5	chr13	112,759,112	
PS_128-CG6	chr13	112,759,124	
	chr13	112,759,355	cg15415452
PS_124-CG6	chr13	112,759,701	
PS_124-CG5	chr13	112,759,704	
PS_124-CG4	chr13	112,759,714	
PS_124-CG3	chr13	112,759,719	cg13692446
PS_124-CG2	chr13	112,759,723	
PS_124-CG1	chr13	112,759,725	
PS_123-CG1	chr13	112,759,782	
PS_123-CG2	chr13	112,759,788	

Assay_cg	Chr	Pos	Candid
PS_123-CG3	chr13	112,759,797	
PS_123-CG4	chr13	112,759,799	

Abbreviations: CpG site (cg), Chromosome (Chr), Genomic position (Pos), Candidate site Illumina Chip analysis (Candid)

Supplemental Table S3. Statistical analysis of methylation in paired normal-adjacent and tumor tissues.

Assay	Group1	Group2	N	*p*	p.adj	sig.adj
PS_127-CG1	adN	TU	152	0.000014	0.000014	***
PS_127-CG2	adN	TU	152	0.000000	0.000000	***
PS_127-CG3	adN	TU	152	0.000001	0.000004	***
PS_127-CG4	adN	TU	152	0.000003	0.000006	***
PS_127-CG5	adN	TU	152	0.000000	0.000000	***
PS_127-CG6	adN	TU	152	0.000000	0.000000	***
PS_127-CG7	adN	TU	152	0.000000	0.000000	***
PS_128-CG1	adN	TU	143	0.000000	0.000000	***
PS_128-CG2	adN	TU	143	0.000000	0.000000	***
PS_128-CG3	adN	TU	143	0.000000	0.000000	***
PS_128-CG4	adN	TU	143	0.000000	0.000000	***
PS_128-CG5	adN	TU	143	0.000000	0.000000	***
PS_128-CG6	adN	TU	143	0.000000	0.000000	***
PS_124-CG6	adN	TU	141	0.000000	0.000000	***
PS_124-CG5	adN	TU	141	0.000000	0.000000	***
PS_124-CG4	adN	TU	141	0.000000	0.000000	***
PS_124-CG3	adN	TU	141	0.000000	0.000000	***
PS_124-CG2	adN	TU	141	0.000000	0.000000	***
PS_124-CG1	adN	TU	141	0.000000	0.000000	***
PS_123-CG1	adN	TU	151	0.000000	0.000000	***
PS_123-CG2	adN	TU	151	0.000000	0.000000	***
PS_123-CG3	adN	TU	151	0.000000	0.000001	***
PS_123-CG4	adN	TU	151	0.000000	0.000001	***

Abbreviations: Number of tissue samples (N), *p*-value (p), adjusted *p*-value (p.adj), significance adjusted (sig.adj), normal-adjacent (adN), tumor tissue (TU), ***: $p < 0.001$. Order of analyzed CpG sites follow genomic positions.

Supplemental Table S4. Cohen´s d analysis of paired normal-adjacent and tumor tissues.

Assay	Cohens_d	conf.low	conf.high	magnitude
PS_127-CG1	-0.36	-0.63	-0.20	small
PS_127-CG2	-0.52	-0.73	-0.36	moderate
PS_127-CG3	-0.41	-0.60	-0.15	small
PS_127-CG4	-0.39	-0.57	-0.24	small
PS_127-CG5	-0.52	-0.76	-0.38	moderate
PS_127-CG6	-0.57	-0.82	-0.37	moderate
PS_127-CG7	-0.47	-0.68	-0.27	small
PS_128-CG1	-0.94	-1.14	-0.72	large
PS_128-CG2	-0.77	-0.92	-0.62	moderate
PS_128-CG3	-0.88	-1.04	-0.74	large
PS_128-CG4	-1.00	-1.21	-0.74	large
PS_128-CG5	-0.70	-0.82	-0.56	moderate
PS_128-CG6	-0.71	-0.84	-0.54	moderate
PS_124-CG6	-0.72	-0.82	-0.58	moderate
PS_124-CG5	-0.75	-0.94	-0.59	moderate
PS_124-CG4	-0.94	-1.13	-0.75	large
PS_124-CG3	-0.99	-1.26	-0.80	large
PS_124-CG2	-0.74	-0.85	-0.63	moderate
PS_124-CG1	-0.69	-0.79	-0.57	moderate
PS_123-CG1	-0.64	-0.86	-0.47	moderate
PS_123-CG2	-0.58	-0.80	-0.43	moderate
PS_123-CG3	-0.44	-0.67	-0.24	small
PS_123-CG4	-0.44	-0.65	-0.28	small

Abbreviations: lower confidence interval (conf.low), upper confidence interval (conf.high)

Supplemental Table S5. Logistic regression analysis of primary RCC with and without distant metastasis.

Var	OR	conf.low	conf.high	*p*.value	sig	*p*.adj
PS_127-CG1	1.01	0.98	1.03	0.477229	.	0.5776984381
PS_127-CG2	1.02	1.00	1.04	0.070338	.	0.1244434455
PS_127-CG3	1.02	0.99	1.04	0.161587	.	0.2186176692
PS_127-CG4	1.02	0.99	1.04	0.150035	.	0.2156746695
PS_127-CG5	1.01	0.99	1.04	0.205768	.	0.2629254691
PS_127-CG6	1.02	1.00	1.04	0.104714	.	0.1720301289
PS_127-CG7	1.02	0.99	1.05	0.137203	.	0.2103780139
PS_128-CG1	1.06	1.03	1.09	0.000011	***	0.0001419599
PS_128-CG2	1.04	1.02	1.07	0.001026	**	0.0021443578
PS_128-CG3	1.04	1.02	1.06	0.000616	***	0.0015750286
PS_128-CG4	1.05	1.02	1.08	0.000169	***	0.0005556692
PS_128-CG5	1.04	1.01	1.06	0.001586	**	0.0030397410
PS_128-CG6	1.04	1.02	1.07	0.000908	***	0.0020875419
PS_124-CG6	1.07	1.04	1.11	0.000019	***	0.0001419599
PS_124-CG5	1.07	1.04	1.10	0.000023	***	0.0001419599
PS_124-CG4	1.06	1.03	1.09	0.000025	***	0.0001419599
PS_124-CG3	1.04	1.02	1.07	0.000342	***	0.0009842636
PS_124-CG2	1.05	1.03	1.08	0.000145	***	0.0005556692
PS_124-CG1	1.07	1.04	1.10	0.000038	***	0.0001746379
PS_123-CG1	1.00	0.98	1.02	0.827579	.	0.8841877577
PS_123-CG2	1.01	0.99	1.02	0.590553	.	0.6791354962
PS_123-CG3	1.00	0.98	1.02	0.845745	.	0.8841877577
PS_123-CG4	1.00	0.97	1.03	0.970453	.	0.9704533482

Abbreviations: variable (Var), odds ratio (OR), lower confidence interval (conf.low), upper confidence interval (conf.high), *p*-value (p.value), significance (sig), Benjamini-Hochberg adjusted p-Value (p.value.adj). Graphical presentation of significant levels: *: $p < 0.05$; **: $p < 0.01$; ***: $p < 0.001$; .: not significant.

Supplemental Table S6. Descriptive statistics of primary and RCC metastatic tissue groups.

Var	Tissue	N	mean	sd	ci	median	min	max
PS_127-CG1	M0	156	13.4	15.0	2.4	6.9	0.8	74.2
PS_127-CG1	Mtx	90	26.2	15.4	3.2	26.4	1.6	68.3
PS_127-CG2	M0	156	18.5	16.1	2.5	12.2	1.4	70.3
PS_127-CG2	Mtx	90	34.4	16.4	3.4	34.0	1.2	76.2
PS_127-CG3	M0	156	15.5	15.3	2.4	9.1	0.8	71.8
PS_127-CG3	Mtx	90	30.8	15.8	3.3	31.6	1.0	77.7
PS_127-CG4	M0	156	14.0	15.1	2.4	7.1	0.8	72.0
PS_127-CG4	Mtx	90	29.0	16.7	3.5	28.7	1.1	74.5
PS_127-CG5	M0	156	23.7	16.2	2.6	19.3	3.2	74.8
PS_127-CG5	Mtx	90	36.9	14.8	3.1	36.2	6.9	91.0
PS_127-CG6	M0	156	24.2	16.9	2.7	19.5	4.0	79.2
PS_127-CG6	Mtx	90	40.5	16.1	3.4	40.0	7.8	100.0
PS_127-CG7	M0	156	11.9	10.9	1.7	7.3	0.3	56.4
PS_127-CG7	Mtx	90	21.9	12.0	2.5	21.1	2.5	60.0
PS_128-CG1	M0	153	21.3	16.5	2.6	16.1	1.8	85.7
PS_128-CG1	Mtx	88	34.8	16.2	3.4	34.0	3.5	89.9
PS_128-CG2	M0	153	14.0	14.8	2.4	8.4	1.3	92.5
PS_128-CG2	Mtx	88	26.3	17.2	3.6	23.9	1.8	96.6
PS_128-CG3	M0	153	18.9	17.0	2.7	11.1	0.8	77.8
PS_128-CG3	Mtx	88	32.9	17.6	3.7	31.3	3.2	97.0
PS_128-CG4	M0	153	22.0	16.3	2.6	14.9	2.0	79.6
PS_128-CG4	Mtx	88	35.3	17.0	3.6	32.3	2.4	93.3
PS_128-CG5	M0	153	12.5	13.9	2.2	6.9	1.1	68.9
PS_128-CG5	Mtx	88	23.8	16.3	3.5	19.5	1.9	95.8

Var	Tissue	N	mean	sd	ci	median	min	max
PS_128-CG6	M0	153	12.3	13.2	2.1	7.2	1.6	81.3
PS_128-CG6	Mtx	88	22.4	17.1	3.6	17.9	2.5	100.0
PS_124-CG6	M0	151	13.5	12.8	2.0	8.2	1.3	78.8
PS_124-CG6	Mtx	84	21.0	14.2	3.1	19.9	0.9	59.7
PS_124-CG5	M0	151	19.6	14.4	2.3	14.4	2.3	71.0
PS_124-CG5	Mtx	84	25.9	15.9	3.4	23.3	1.8	78.4
PS_124-CG4	M0	151	23.0	16.7	2.7	17.0	1.8	80.5
PS_124-CG4	Mtx	84	31.6	16.8	3.6	29.2	1.6	88.7
PS_124-CG3	M0	151	28.3	18.5	3.0	22.6	4.7	92.0
PS_124-CG3	Mtx	84	34.5	15.5	3.4	34.9	1.5	82.9
PS_124-CG2	M0	151	12.3	12.9	2.1	7.5	1.3	63.0
PS_124-CG2	Mtx	84	20.8	14.3	3.1	17.4	1.0	63.2
PS_124-CG1	M0	151	9.5	10.9	1.8	4.7	0.8	57.3
PS_124-CG1	Mtx	84	19.0	14.0	3.0	16.7	0.8	76.8
PS_123-CG1	M0	156	30.2	20.7	3.3	22.6	1.8	93.8
PS_123-CG1	Mtx	85	54.5	19.3	4.2	57.3	4.8	97.5
PS_123-CG2	M0	156	24.9	19.9	3.2	16.4	3.4	92.7
PS_123-CG2	Mtx	85	47.7	20.1	4.3	48.4	2.4	94.4
PS_123-CG3	M0	156	17.2	17.4	2.8	10.8	2.5	90.3
PS_123-CG3	Mtx	85	37.5	21.1	4.6	37.3	4.4	92.8
PS_123-CG4	M0	156	13.5	14.7	2.3	7.7	0.9	84.0
PS_123-CG4	Mtx	85	31.3	19.6	4.2	30.0	1.9	77.4

Abbreviations: standard deviation (sd), confidence interval (ci), minimum (min), maximum (max), primary RCC tissue without distant metastasis (M0), metastatic tissue (Mtx).
