# Supplementary material for: Metastasis-Specific CpG Island DNA Hypermethylation of the Long Non-Coding RNA Gene 00404 in Renal Cell Carcinoma
Source: Cancers (Basel). 2025 Jun 30;17(13):2204. doi: 10.3390/cancers17132204 (PMC12249281; doi:10.3390/cancers17132204)
Supplement: Supplementary file 1 [file cancers-17-02204-s001.zip › Figures.pdf]

Supplemental Figure S1. Genomic context of the LINC00404 and LINC00403 genes, location of the CpG islands, the approximate location of candidate CpG sites, and DNA methylation of specific CpG sites in cancer cell line models as reported by the ENCODE project.

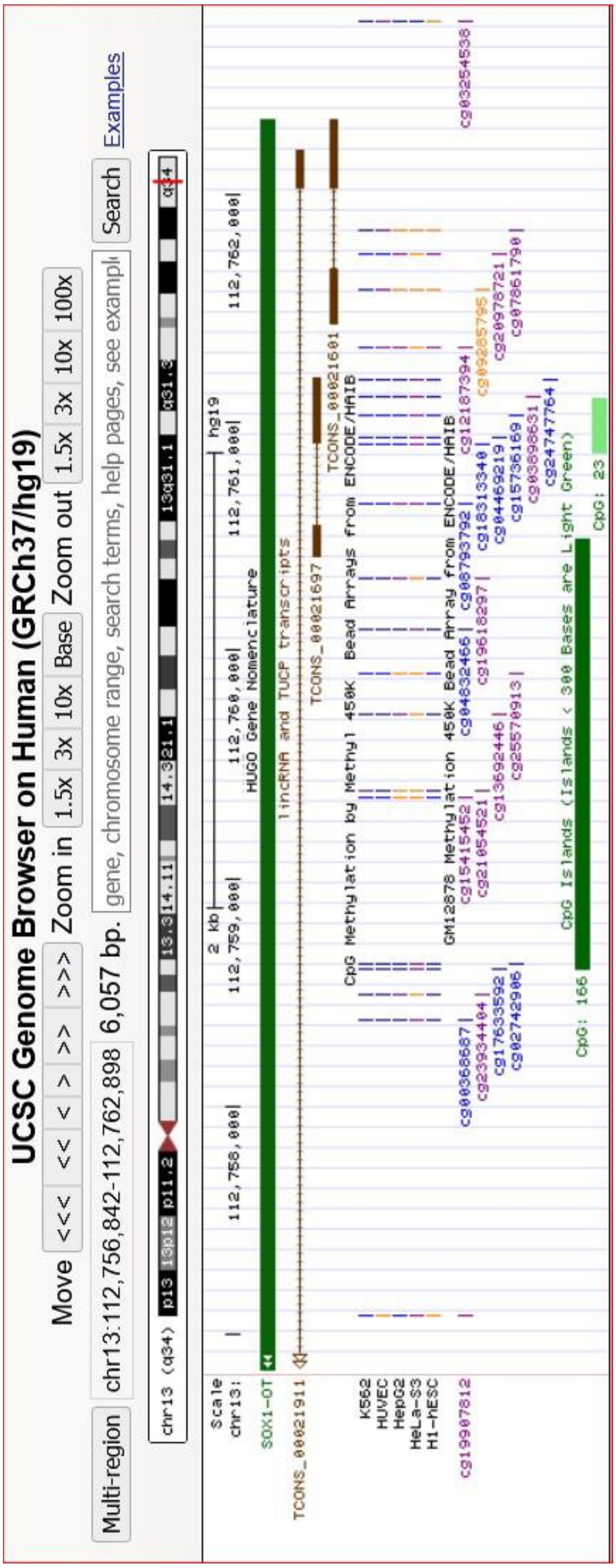

Supplemental Figure S2. Box plot analysis of tumor group comparisons across all CpG sites following dichotomization: (**A**) presence or absence of distant metastasis (M), (**B**) tumor stage classified as high versus low (T), (**C**) tumor grade classified as high versus low differentiation (G), and (**D**) histological subtype, distinguishing papillary or clear cell, including mixed tumor histologies.

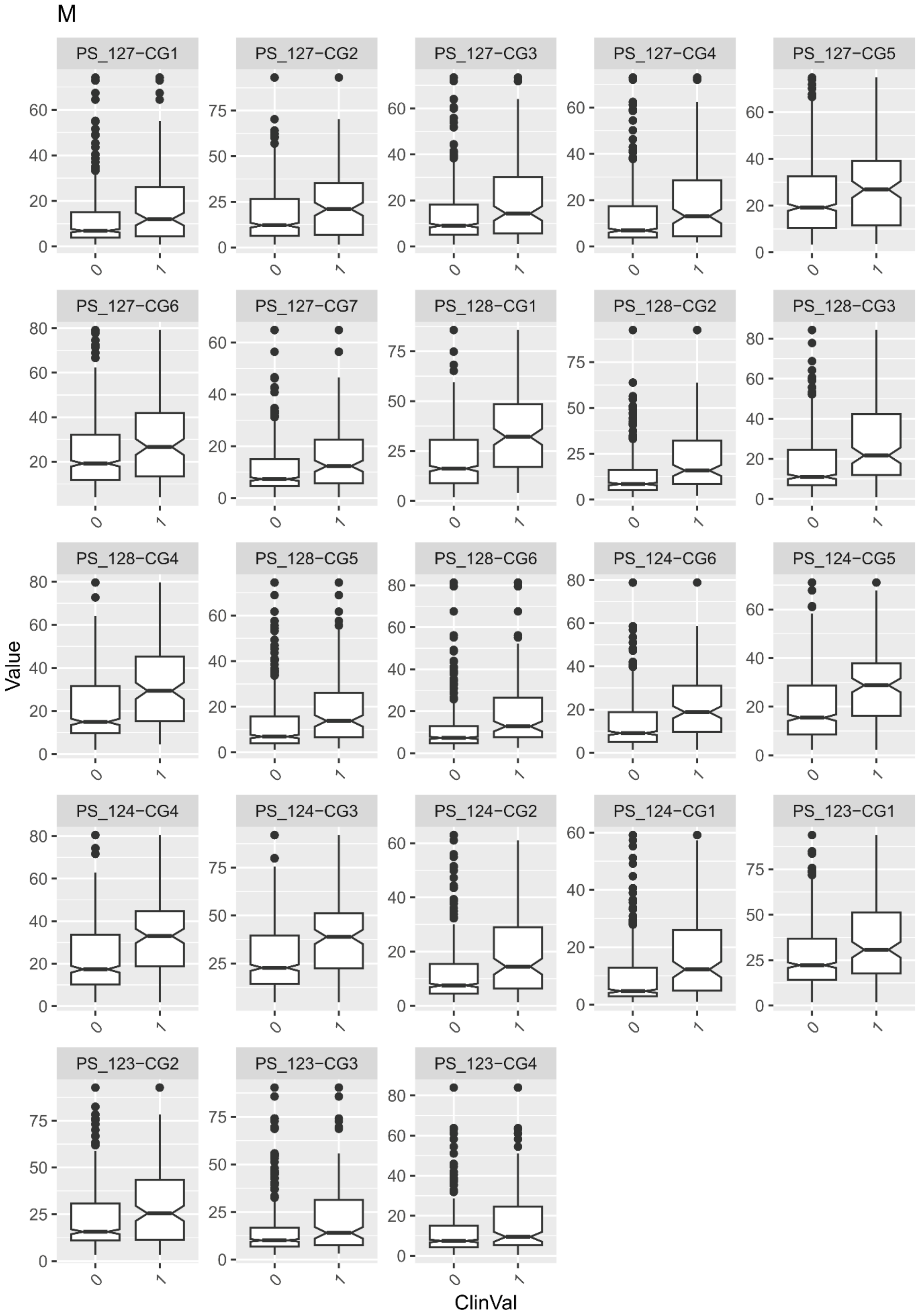
M
PS_127−CG1
PS_127−CG2
PS_127−CG3
PS_127−CG4
PS_127−CG5
PS_127−CG6
PS_127−CG7
PS_128−CG1
PS_128−CG2
PS_128−CG3
PS_128−CG4
PS_128−CG5
PS_128−CG6
PS_124−CG6
PS_124−CG5
PS_124−CG4
PS_124−CG3
PS_124−CG2
PS_124−CG1
PS_123−CG1
PS_123−CG2
PS_123−CG3
PS_123−CG4
Value
ClinVal

(**A**)

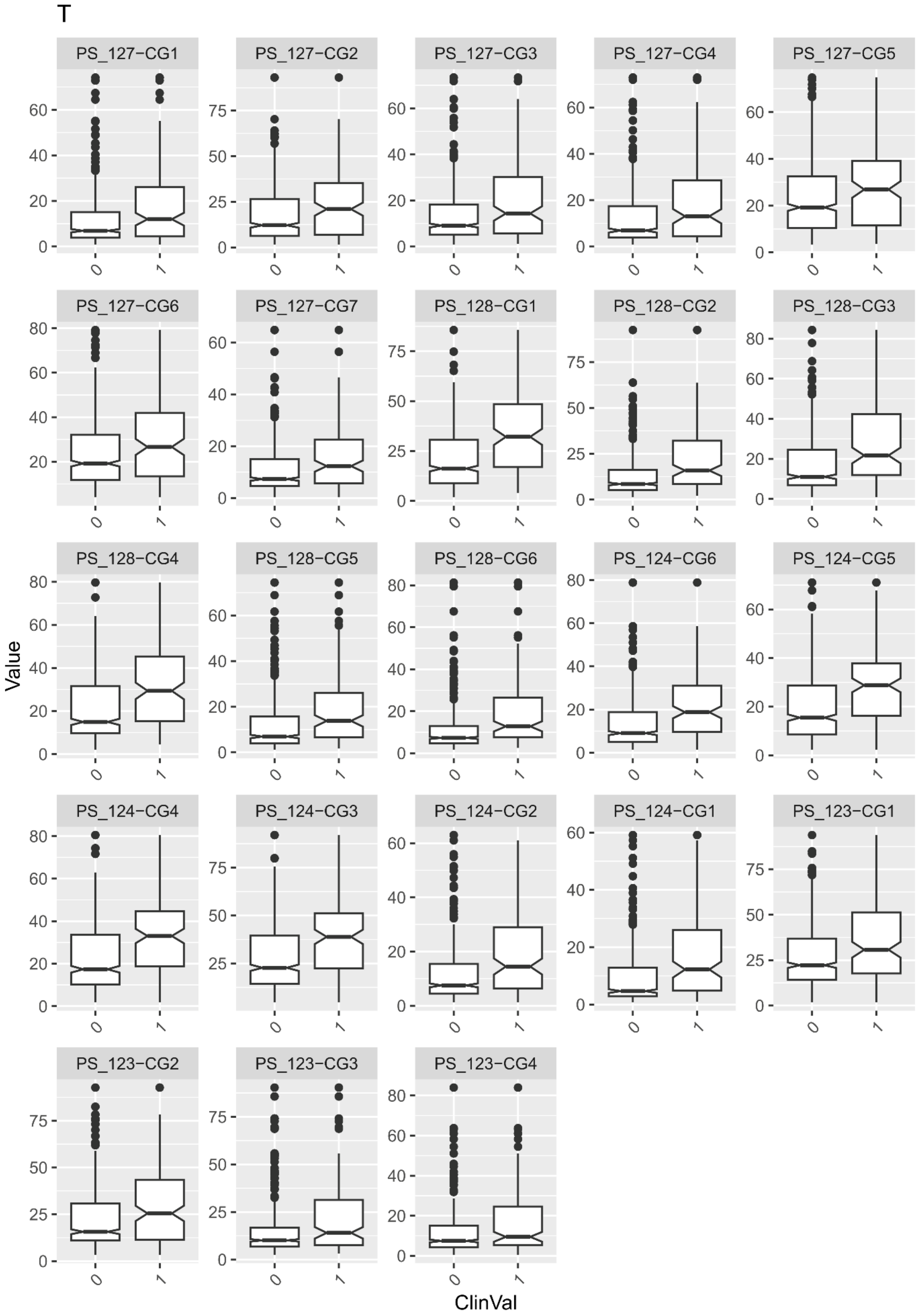
T
PS_127−CG1
PS_127−CG2
PS_127−CG3
PS_127−CG4
PS_127−CG5
PS_127−CG6
PS_127−CG7
PS_128−CG1
PS_128−CG2
PS_128−CG3
PS_128−CG4
PS_128−CG5
PS_128−CG6
PS_124−CG6
PS_124−CG5
PS_124−CG4
PS_124−CG3
PS_124−CG2
PS_124−CG1
PS_123−CG1
PS_123−CG2
PS_123−CG3
PS_123−CG4
Value
ClinVal

(B)

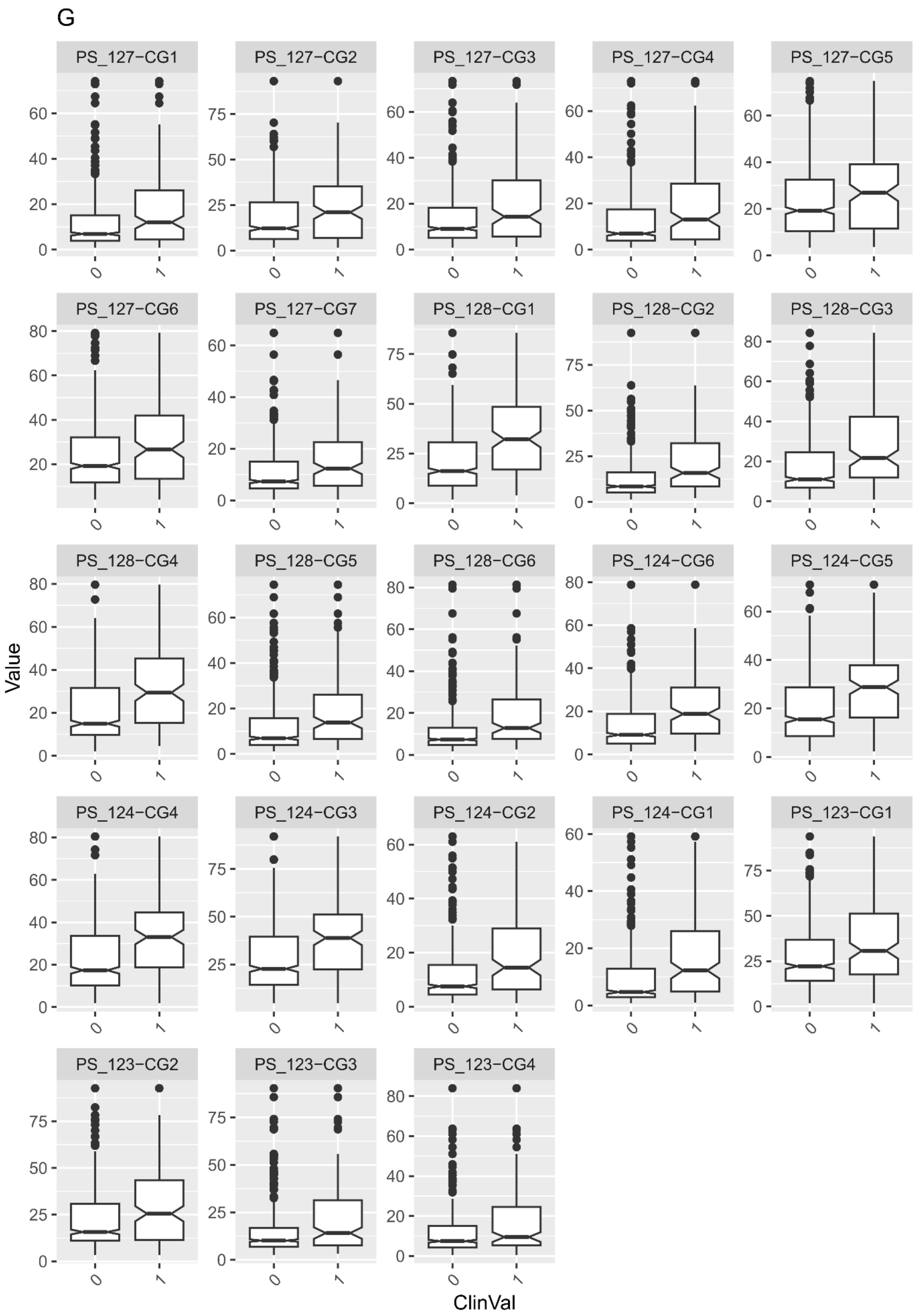
G
PS_127−CG1
PS_127−CG2
PS_127−CG3
PS_127−CG4
PS_127−CG5
PS_127−CG6
PS_127−CG7
PS_128−CG1
PS_128−CG2
PS_128−CG3
PS_128−CG4
PS_128−CG5
PS_128−CG6
PS_124−CG6
PS_124−CG5
PS_124−CG4
PS_124−CG3
PS_124−CG2
PS_124−CG1
PS_123−CG1
PS_123−CG2
PS_123−CG3
PS_123−CG4
Value
ClinVal

(C)

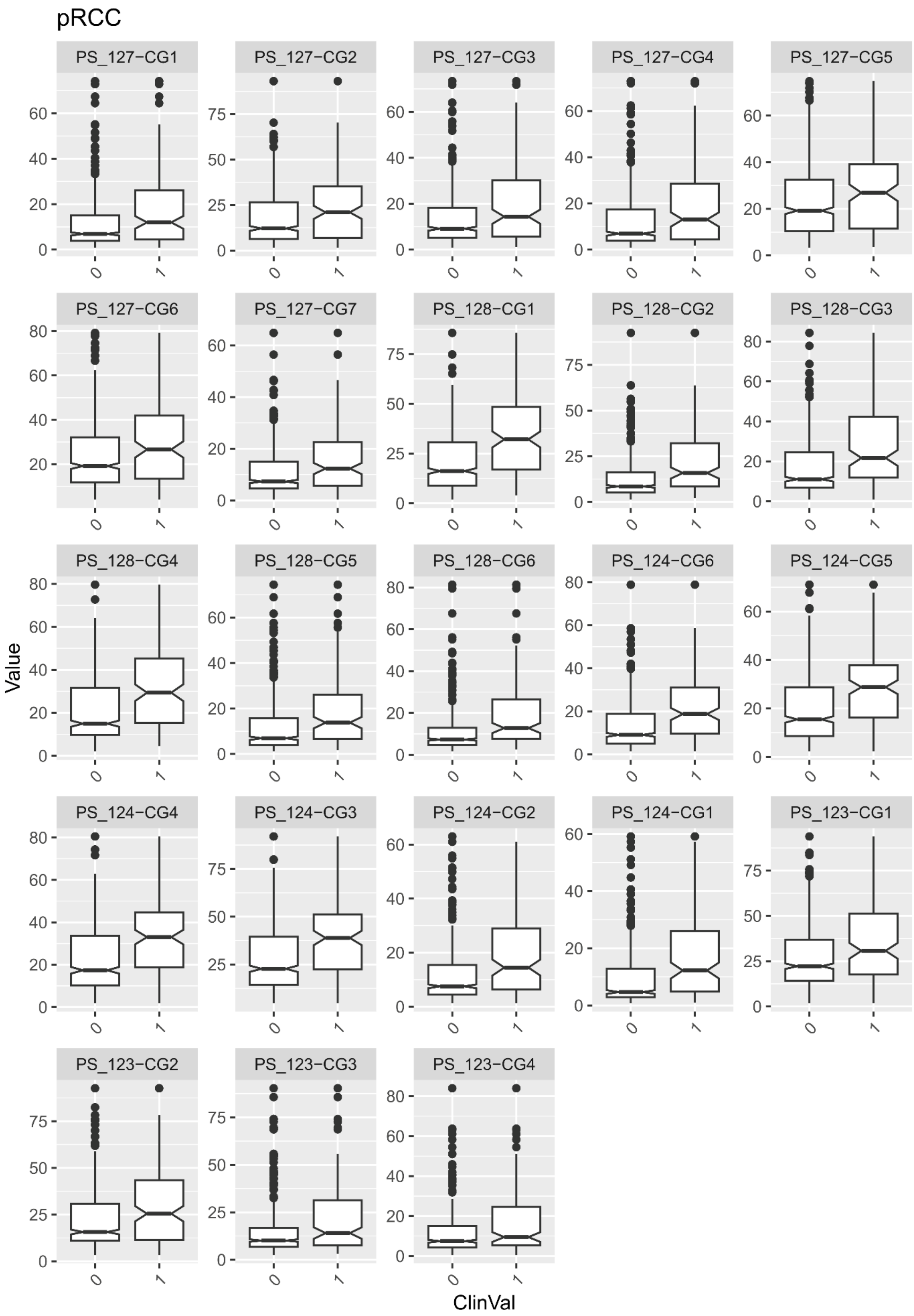
pRCC
PS_127−CG1
PS_127−CG2
PS_127−CG3
PS_127−CG4
PS_127−CG5
PS_127−CG6
PS_127−CG7
PS_128−CG1
PS_128−CG2
PS_128−CG3
PS_128−CG4
PS_128−CG5
PS_128−CG6
PS_124−CG6
PS_124−CG5
PS_124−CG4
PS_124−CG3
PS_124−CG2
PS_124−CG1
PS_123−CG1
PS_123−CG2
PS_123−CG3
PS_123−CG4
Value
ClinVal

(D)
